# Supplementary figures and images for: Identification of an evolutionarily conserved domain in Neurod1 favouring enteroendocrine versus goblet cell fate
Source: PLoS Genet. 2022 Mar 14;18(3):e1010109. doi: 10.1371/journal.pgen.1010109 (PMC8959185; doi:10.1371/journal.pgen.1010109)

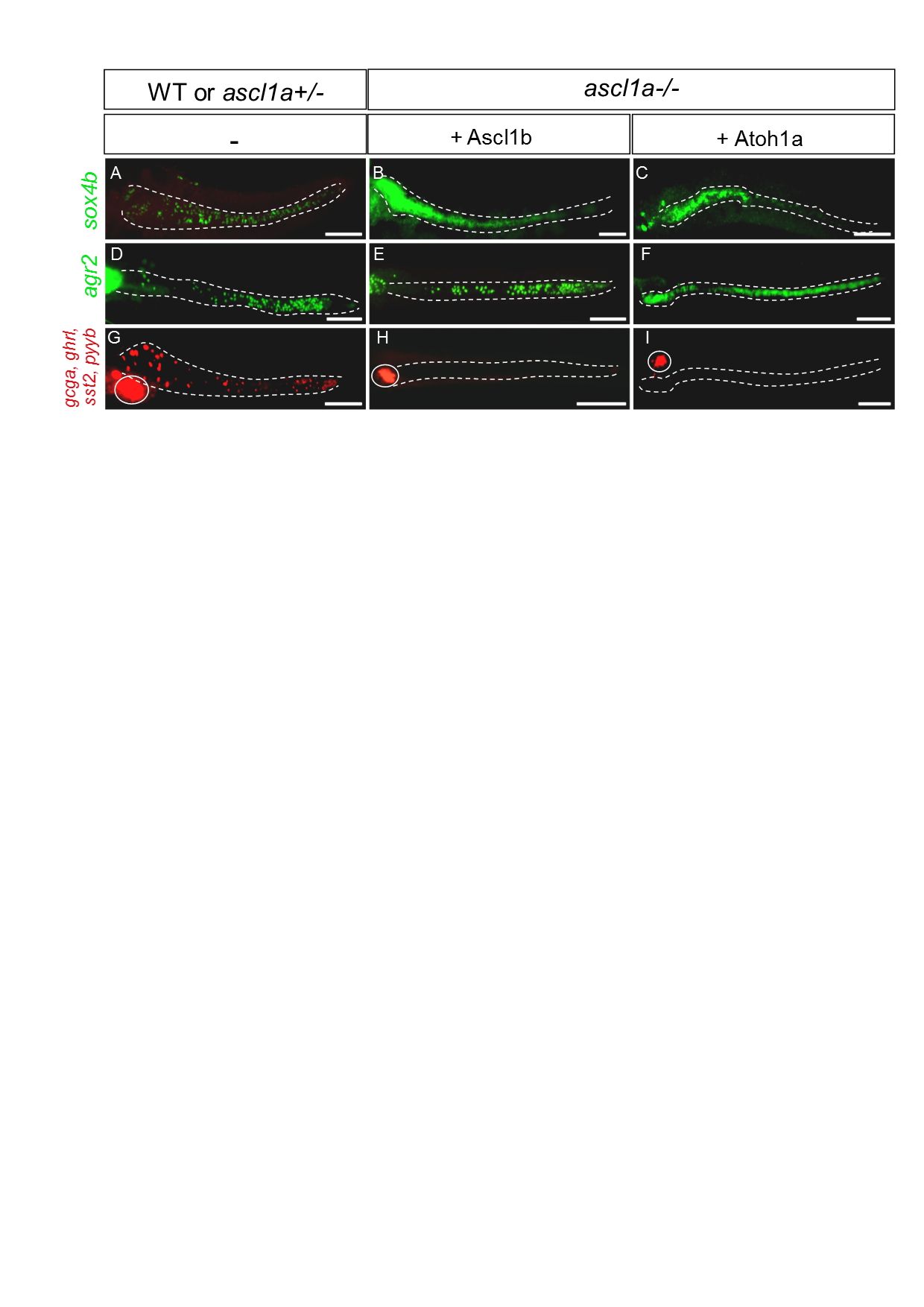

Supplement: S1 Fig — A-C: FISH performed at 55 hpf with a sox4b probe on ascl1a-/- or control sibling embryos heat-shocked at 36 and 46 hpf. D-I: FISH performed at 96 hpf with the agr2 probe (D-F) and a mix of hormone probes (ghrelin (ghrl), peptide YYb (pyyb), glucagon-a (gcga) and somatostatin-2 (sst2)) (G-I) on ascl1a-/- or control sibling embryos heat-shocked at 36, 46 and 56 hpf. The transgenic line used is indicated on the left part of the figure as well as the genotype of the larvae; the ascl1a-/- larvae were identified by the loss of the pituitary prl expression (not shown). The pancreas is encircled while the location of gut, visualised with a DAPI staining (not shown), is delimited by dashed lines. All Views are ventral with the anterior part to the left. Scale bar: 100 μm. (TIF) [file pgen.1010109.s001.tif]

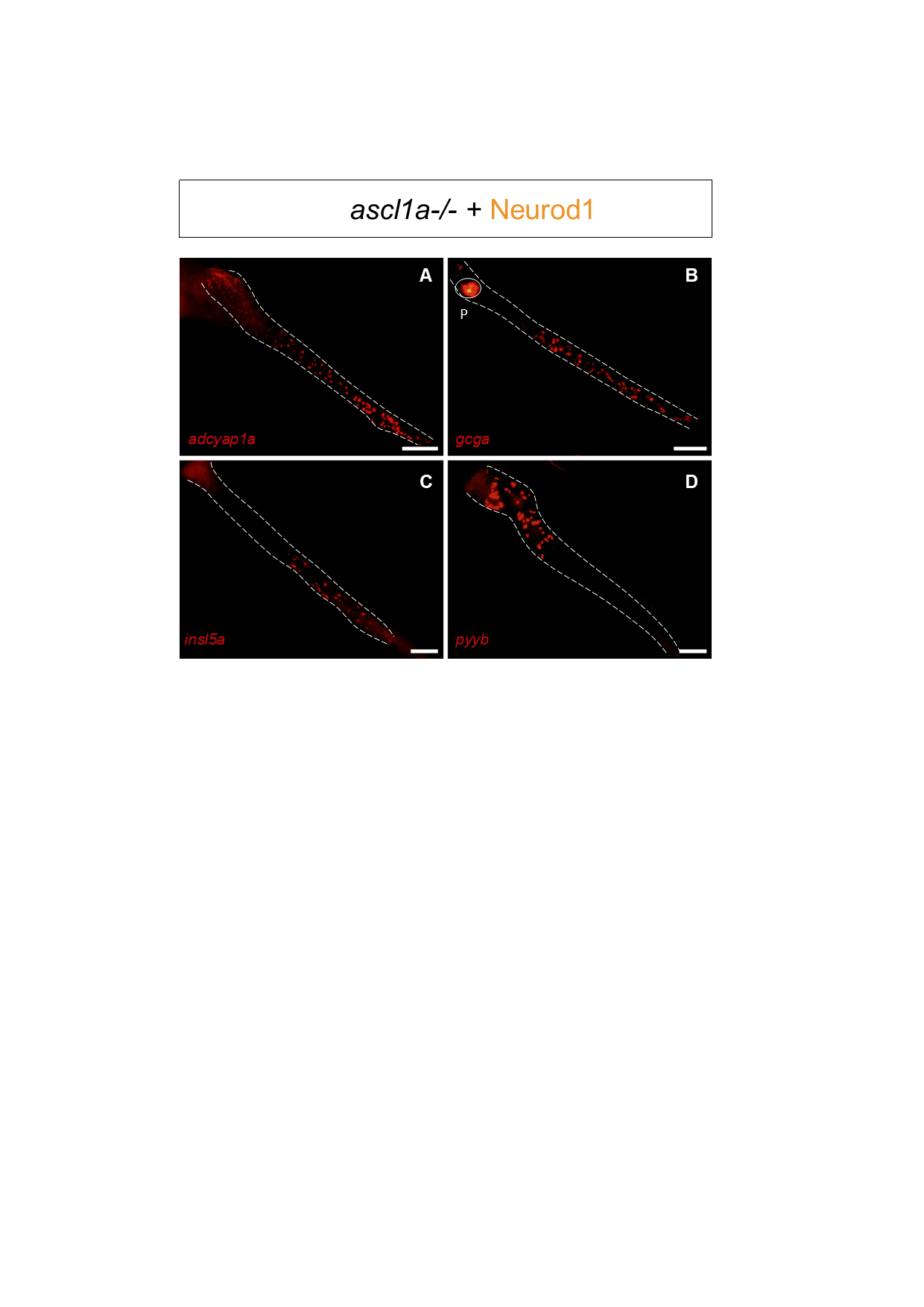

Supplement: S2 Fig — A-D: FISH performed at 96hpf with adcyap1a (A), gcga (B), insl5a (C) and pyyb (D) probes on ascl1a-/-; Tg(hsp70l:eGFP-2A-Neurod1) larvae heat-shocked at 36, 46 and 56hpf. The ascl1a-/- larvae (not shown) do not express any hormones [5]. All views acquired with a fluorescence microscope are ventral with the anterior part to the left. The pancreas is encircled while the location of gut, visualised with a DAPI staining (not shown), is delimited by dashed lines. Scale bar: 100μm. (TIF) [file pgen.1010109.s002.tif]

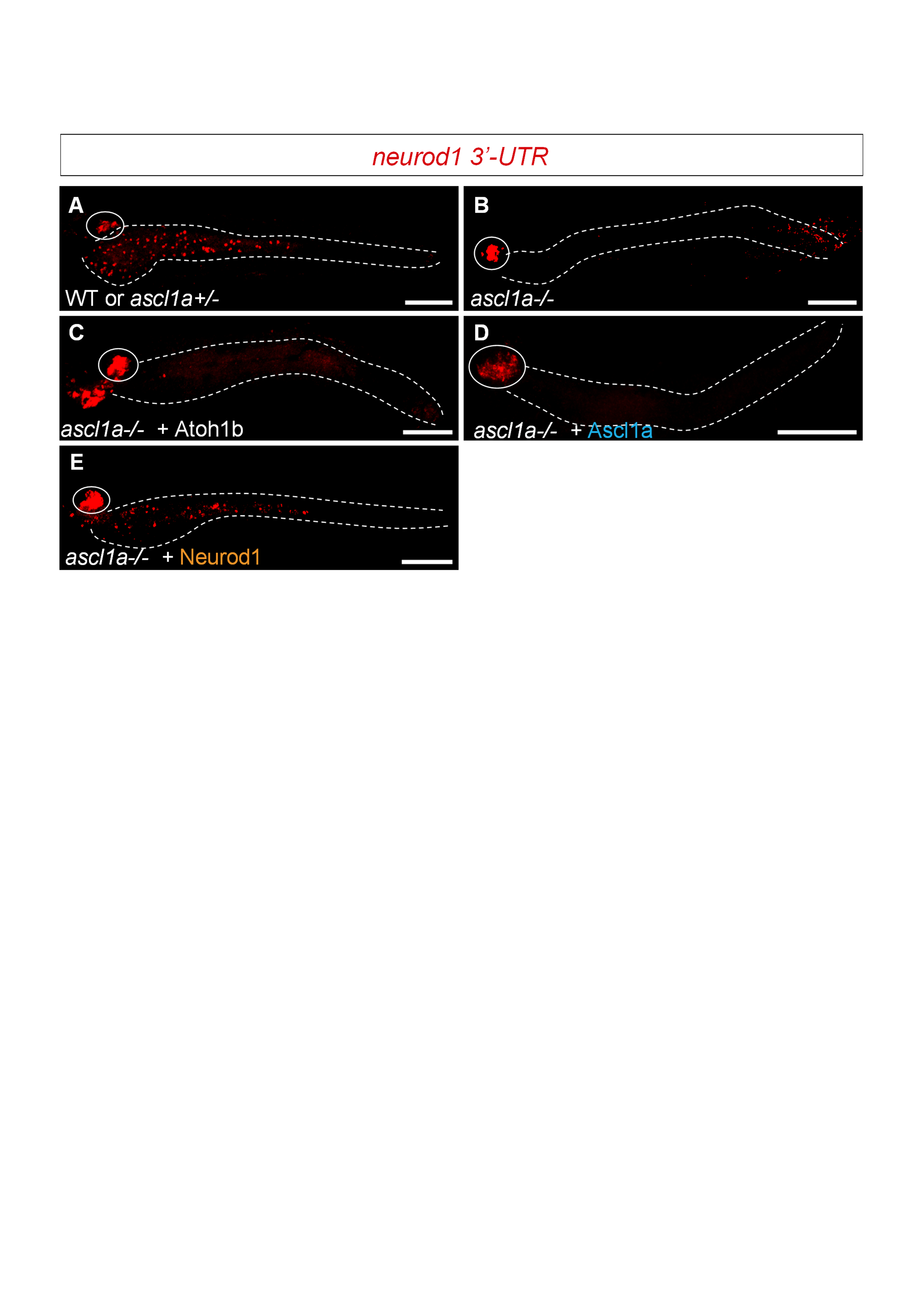

Supplement: S3 Fig — FISH performed at 72hpf with a 3’UTR neurod1 probe on wild-type (A), ascl1a-/- (B), ascl1a-/-; Tg(hsp70l:atoh1b-Myc) (C) ascl1a-/-; Tg(hsp70l:eGFP-2A-ascl1a) (D) and ascl1a-/-; Tg(hsp70l:eGFP-2A-neurod1) (E) larvae, heat-shocked at 36, 46 and 56hpf. All views are ventral with the anterior part to the left and represent confocal projection images. The pancreas is encircled while the location of gut, visualised with a DAPI staining (not shown), is delimited by dashed lines. Scale bar: 100μm. (TIF) [file pgen.1010109.s003.tif]

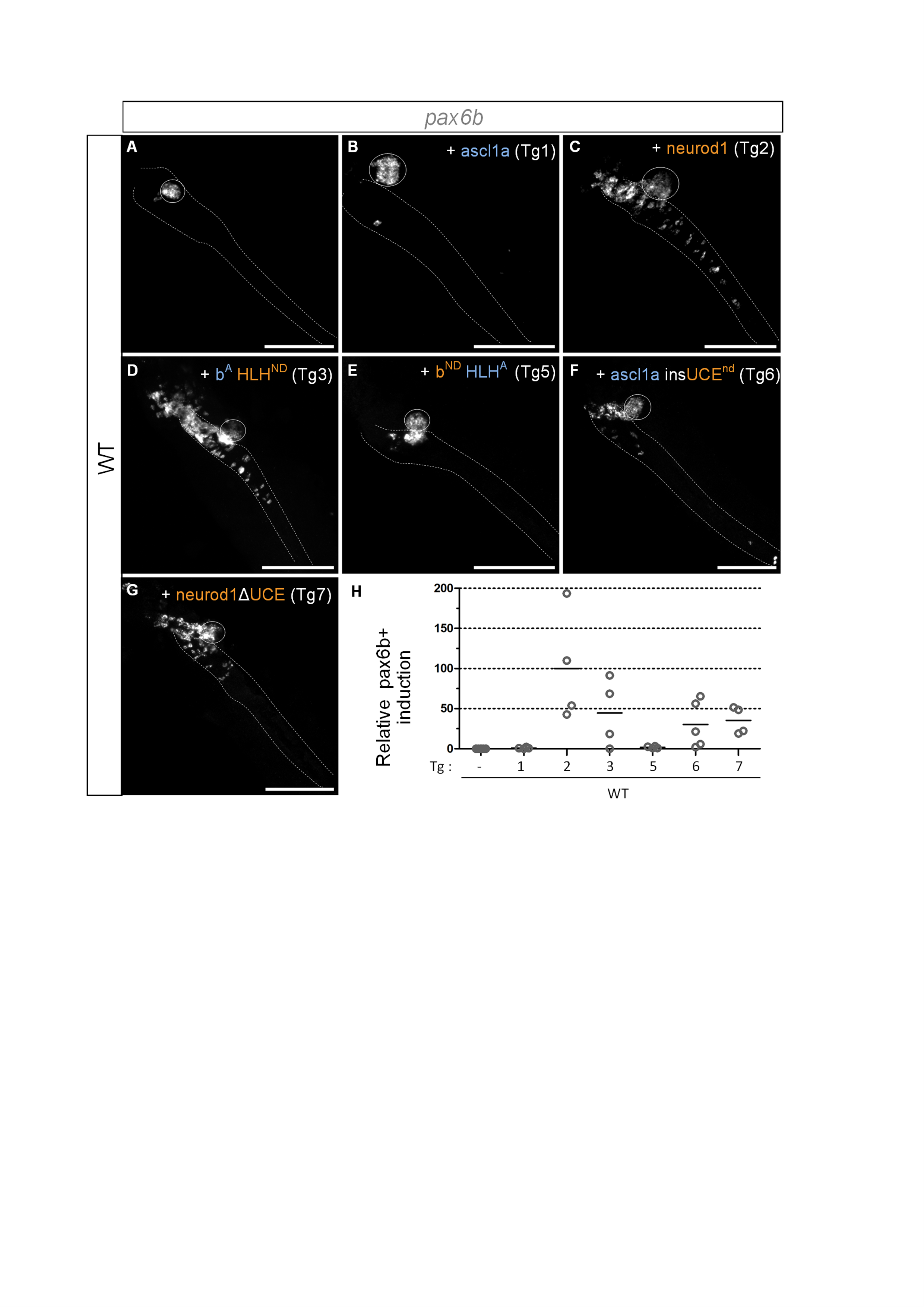

Supplement: S4 Fig — A-G: FISH performed at 56 hpf with the pax6b probe on wild-type embryos without (A) or with the transgenes indicated on the Fig (B-G) and heat-shocked at 38 and 48 hpf. H: Quantification using Imaris software of the volume occupied by the induced pax6b+ cells in the gut for the conditions A to G with Tg2 (Neurod1), arbitrarily set to 100%. All Views are ventral with the anterior part to the left and represent confocal projection images. The pancreas is encircled while the location of the gut, visualised with a DAPI staining (not shown), is delimited by dashed lines. Scale bar: 100μm. (TIF) [file pgen.1010109.s004.tif]

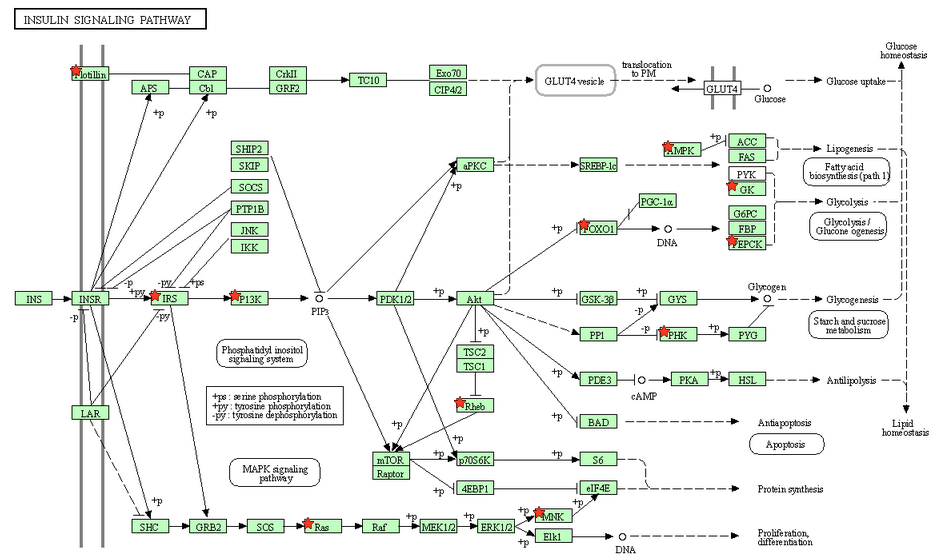

Supplement: S5 Fig — Gene Ontology analysis highlights significant enrichment for insulin signaling pathway in neurod1ΔUCE DE genes. 13 out of 60 genes of the cascade are differentially expressed (indicated by red dots). (TIF) [file pgen.1010109.s005.tif]
