# Supplementary material for: Identification of an evolutionarily conserved domain in Neurod1 favouring enteroendocrine versus goblet cell fate
Source: PLoS Genet. 2022 Mar 14;18(3):e1010109. doi: 10.1371/journal.pgen.1010109 (PMC8959185; doi:10.1371/journal.pgen.1010109)
Supplement: Attachment — Submitted filename: 2022 01 04 reponses to the reviewers.docx [file pgen.1010109.s011.docx]

Dear Editor and Reviewers,

Please find enclosed our revised manuscript entitled “Identification of an evolutionarily conserved domain in Neurod1 essential for triggering enteroendocrine cell differentiation” for further consideration at PLOS Genetics. We present now a revised manuscript with most of the points that had to be corrected. Importantly, we address the main concern of the editors and of the reviewer 2 which was the lack of data describing the phenotype of *neurod1*-null mutant larvae precluding definitive conclusions on the role of this TF. The *neurod1* null mutant displays very similar intestinal defects as the *neurod1^ΔUCE^* mutant supporting the crucial function of the UCE domain for Neurod1 activity. An additional paragraph and a new figure has been therefore added to the paper. We would like also to modify slightly the title: “Identification of an evolutionarily conserved domain in Neurod1 favouring enteroendocrine versus goblet cell fate” that, we believe, reflects better the message of the paper.

**Comments to the Authors:**

Reviewer #1: The authors investigate whether different members of the achaete-scute family of basic helix loop helix transcription factors are able to restore loss of secretory cells in ascl1a mutants. They find that all members of the family are able to rescue secretory cells. Each family member does not rescue all classes secretory cells. Neurod1 is the is the only family member able to rescue enteroendocrine cells. The other family members rescue goblet cells but not enteroendocrine. Neurod1 has a conserved region that is able to induce enteroendocrine fates and repress goblet cell fate. Transcriptomics of ectopically expressed neurod1 and varients upregulate a group of differentially expressed genes and ascl1a downregulates many of these genes. There is a common group of differentially expressed genes shared by neruod1 and ascl1a.

The authors do a good job on analysis of the activity of ectopically expressed achaete-scute family members. The activity of each of these family members is nicely characterized in terms of secretory cell formation and differential gene expression.

Regarding ectopic expression of achaete-scute family members, the authors demonstrate misexpression of secretory cell markers and genes within the intestine but do not perform histology to show whether ectopic expression alters the overall structure of the embryo and more specifically the structure of the intestine. Authors should show images of whole embryo and histology of intestine to demonstrate whether structure is normal other than changes in secretory cell content.

As required, we performed microtome serial sections of 5 dpf Neurod1^ΔUCE^ mutant and control larvae and stained successive sections with haematoxylin/eosin or with eosin/alcian blue to visualise the goblet cells. We found a widening of the midgut region with an increased amount of goblet cells in this region. In contrast, we did not notice other defects in the other regions of the intestine. This new result that confirms the WGA immunohistochemistry has been added in figure 8.

Authors should more specifically describe controls for heat shock experiments. It was hard to find description of controls in either in results or material and methods.

We have now added more precisely in the legend of each figure which controls were used.

Reviewer #2: In the present manuscript, Reuter et al address the specificity of action of a family of closely related basic Helix-Loop-Helix (bHLH) containing transcription factors in the establishment of secretory lineages in the developing zebrafish larval gut. To achieve this, the authors use a rescue approach based on a series of global misexpression transgenic constructs in a mutant background, ascl1a/pia loss-of-function, that abrogates all secretory linages. In this manner, they find that while Ascl1a/b, Atoh1a/b and Neurod1 factors all drive cells in the gut to differentiate, the specific identity of the neurons that are generated varies depending on the factor used – the former two families rescue goblet cells and Neurod1 rescues enteroendocrine (EE) cells. Using a subsequent series of transgenes driving chimeric bHLH factors, the authors identify a region of Neurod1 responsible for its EE cell specificity. Deletion of this region using Crispr/Cas9 genome editing partially abrogates this specific function. Finally, a series of transcriptome analyses highlights potential target lists that are downstream of these bHLH factors.

Overall, the manuscript presents a considerable sum of data, including RNAseq data. Unfortunately, the relatively simple message the authors try to convey via the title or abstract is not completely supported.

My major and minor comments are as follows:

Major comments:

The authors construct a gene regulatory pathway suggesting that Ascl1a lies upstream of Neurod1, which in turn inhibits Gfi1aa leading to inhibition of goblet cell fate, and that this pathway hinges on the presents of the Ultra-conserved element (UCE) in Neurod1. Unfortunately, the evidence for this is largely anecdotal and is not directly tested.

1) To start, the inability of Ascl1a misexpression to completely rescue the ascl1a mutant phenotype is perturbing. The authors present various arguments as to why this might be the case. Nonetheless, it severely biases the possible interpretations of the rescue results for Neurod1 as it begs the question of whether what neurod1 can do in misexpression reflects its true function – Neurod1 can do certain things whether it does them endogenously is unclear.

The question “How related factors like the ARP/Ascl factors retain their specificity of activity, especially when they recognize the same consensus DNA motif” is not yet understood [1] . This specificity could be due either to the cellular environment in which they are endogenously expressed, or due to intrinsic properties of the factors themselves. To answer this important question, these factors have to be expressed in the same environment and, therefore, many studies use GOF to tackle this aspect that cannot be studied by LOF experiments. For example, to understand how ASCL1, ASCL2, and MYOD1, that recognize the same consensus DNA motifs but bind largely different genomic sites in vivo, Casey et al **ectopically expressed these factors in embryonic stem cells** and show that these factors retain distinct binding sites [1]. This indicates that the specificity of binding is an intrinsic property of the factors. This finding published in Genome research could not be discovered by LOF. Indeed, LOF does not allow to compare the **intrinsic properties of related factors** when these factors are expressed in different tissues. Many other studies have published important data based on GOF [2] and for example the activity of proneural genes have been extensively studied by GOF as reviewed by ([3]

In this study, GOF experiments allowed us to identify the UCE domain which is required to repress the goblet cell lineage. This important finding is **confirmed in LOF** as we detect an increase of goblet cells concomitant to a reduction of EEC in the *neurod1^ΔUCE^* and in the neurod1 null mutants. The identification of this important conserved domain would not have been possible by LOF, therefore **validating the pertinence of performing GOF**.

2) The phenotype of neurod1-null mutant larvae needs to be presented. While the UCE mutant established by the authors is intriguing, without the neurod1-null, it is not easy to interpret the UCE-/- phenotype. If the null and UCE mutations show the same phenotype, for instance, it becomes difficult to conclude a specific function for the UCE.

As requested, we present now the phenotype of the neurod null mutant and an additional paragraph and a new figure 11 has been added to the paper. Briefly, we found a very similar intestinal phenotype of *neurod1^-/-^* mutant compared to *neurod1^ΔUCE^***^,^** both showing an increase of goblet cells and a general reduction of EEC. Furthermore, similar subsets of EEC are affected as 7 out of the 10 EEC analysed show similar perturbations in *neurod1^-/-^* mutant compared to *neurod1^ΔUCE^*. This indicates that the **UCE domain has a very important role in the neurod1 protein at the level of intestine as the deletion of this domain leads nearly to the same phenotype as a full knock-out.** However, as the null *neurod1^-/-^* mutant is lethal while the *neurod1^ΔUCE^* mutant is viable, this indicates that the UCE domain is not (or less) required for Neurod1 activity in other organs.

3) The authors speculate that the inability of Ascl1a to rescue the EE lineage is due to its inability to restore neurod1 expression. A key element that could have helped the authors arguments would have been to know if misexpression of Neurod1 can rescue in the neurod1-null background.

If I understood correctly the comment, the reviewer asks that we determine whether overexpressing Neurod1 in neurod1-/- would restore EEC while Ascl1a would not. As the phenotype of the neurod1-/- is quite mild compared to the ascl1a-/- phenotype, this experiment has not been performed because it would require extensive quantification of the EE to evaluate their rescue by Neurod1 and Ascl1a. This would require a lot of work and time.

4) The authors speculate about Neurod1/Gfi1aa interactions, but to my knowledge no role for Gfi1aa has been established in the zebrafish larval gut.

We agree with the reviewer that the role of Gfi1aa has not been established in the zebrafish intestine but it has been well established in mice and many factors display similar role in zebrafish and mouse like for example for pax6, nkx6.1, isl1…. . Furthermore, like in mouse, gfi1aa is expressed in goblet cell in zebrafish [4] .

Only 21 genes are induced by Neurod1^ΔUCE^ and Neurod1^ΔECD^ but not by Neurod1 and Gfi1aa is the most significant one with an upregulation of 60- and 38-fold in Neurod1^ΔUCE^ and Neurod1^ΔECD^, respectively. This highly suggests (but do not prove) that the induction of the goblet cells seen in Neurod1^ΔUCE^ and Neurod1^ΔECD^ and not in Neurod1 is the consequence of the induction of gfi1aa. To prove this, we could have analysed the gfi1aa-/- mutant and determined whether the mutant displays a loss of goblet cells. This mutant has been generated, is viable and displays normal haematopoiesis due to compensation by the paraloguous gene gfi1ab. It is therefore likely that the same compensation would occur at the level of the intestine. Compensation is indeed very common in zebrafish due to the presence of paralogous gene or even due to transcriptional adaptation [5] . Due to a lack of time and also because this manuscript presents already a considerable sum of data as stated by the reviewer, we did not generate a double gfi1aa/gfi1ab mutant.

5) The RNAseq data may prove to be a useful resource for future studies. As these datasets are based on misexpression, however, it is difficult to assess whether they reflect the endogenous activities of the various bHLH factors. As these datasets neither support nor contradict the model presented in the abstract, they could be removed without altering the interpretation the authors present concerning the Ascl1a>Neurod1>Gfi1aa pathway.

The RNAseq data performed on GOF revealed notably 104 differentially expressed (DE) genes upon overexpression of Neurod1 compared to control embryos. Among them, **several genes such *pax4*, *rfx6*, *insm1a/b,* genes reported to be direct targets of Neurod1** in human insulinoma cell line [6], **therefore validating our GOF experiments** It also highlights a list of 10 genes involved endocrine pancreas development”. This endocrine gene set includes, in addition to the 3 direct target described above, *pax6b*, *nkx2.2a* (which are involved in EEC differentiation) and *c2cd4a* and *tmsf4a*.

These RNAseq also highlight genes which are induced by Neurod1 and repressed by Ascl1a (Fig 6B, green dots). This is the case notably for *crabp1* and *greb1l*, two important actors for retinoic acid signalling suggesting that RA could have also a positive effect on endocrine cell differentiation in the gut like it is observed for the pancreas.

The RNAseq data also notably revealed that Neurod1^ΔUCE^ (Tg7) and Neurod1^ΔECD^ (Tg8), both are able to induce *gfi1aa* expression and this important finding would not be possible without RNAseq profiling.

Therefore we believe that these RNAseq data are useful resource, important for researchers working on zebrafish gut as it would allow them to know whether their favourite gene is expressed in the gut at 52 hpf or in the EEC at 4 dpf. But also for people working on the nervous system as Neurod 1 is also an important actor for neurogenesis and the list of Neurod1 regulated genes could be of great interest to them.

Minor comments:
1) The distribution of the various markers along the A/P axis of the gut appears to vary as a function of the bHLH used in the rescue experiments. This is more clearly the case for sox4b expression (panels C,D,E). Is this significant?

No, it is not significant. The rescue can be variable from one embryo to the other. What was important here was to determine whether we could see *sox4b* rescue or not. And we found that sox4b was rescued in all embryos tested but the extent of the rescue can vary from one larvae to another.

2) Figure 2 would benefit from having wildtype controls presented as in Figure 1.

As requested, we added the image for the sibling control in figure 2.

3) It is not clear why the activity of Tg4 and 8 were not determined concerning pax6b induction? What does “V” indicate in Table 1?

The V sign means that we had full rescue of sox4b and this information has been added in the legend. Concerning *pax6b* induction, this analysis was done to determine whether RNA-seq profiling on the gut **of wild-type** transgenic embryos instead of ascl1a-/- mutant would allow the identification of genes specifically regulated by Neurod1. We found that the tg2 (Neurod1), tg3, tg6 and tg7 transgenes, able to rescue the EECs, are also able to induce *pax6b* earlier **in wt embryos. In contrast,** tg1 (ascl1a) and tg5, which are not able to rescue EEC, are not able to induce pax6b earlier in wt embryos. Therefore, we had a good correlation between the two tests and we found that it was not necessary to test all the 8 constructs as these experiments take a lot of times. Based on that correlation, we decided to perform RNAseq on wt transgenic embryos.

4) The distinction between full and partial rescue presented in Figure 4F is not specified.

We have now added the following information in the legend : “The larvae expressing *agr2* at a level similar to wt or higher were classified as “Full rescue” while larvae with lower expression of *agr2* compared to wt were classified as “partial rescue”. Larvae without *agr2* expression were classified as “no rescue”.

5) Advancing the time window for the RNAseq analyses such that it precedes the endogenous induction of pax6b does not really “circumvent” the problem that ascl1a mutants cannot be distinguished from wildtype or heterozygote siblings. It does, however, minimize some of the risks inherent in these experiments.

I am sorry but we do not understand this comment. We performed RNAseq at 52 hpf, 4 hours after the heatshock and therefore 8 hours before pax6b endogenous expression as, at this timepoint, Neurod1 direct targets can be identified by RNAseq. We do not see what are the risks mentioned by the reviewer.

6) The images in Figure 6 are not of sufficient quality and should be replaced.

We have changed the figure 6 (now labelled figure 7) with confocal images of fluorescent in situ instead of visible in situ. We also added additional data showing that the Tg8 (Neurod1^ΔECD )^ is also able to induce *gfi1aa* in the gut like Tg7 (Neurod1^ΔUCE^ ). Furthermore, we show that Tg1 (Ascl1a) is not able to induce gfi1aa. All these data validate therefore the RNAseq data.

7) Figure 7 would benefit from high magnification. As cell counts are presented, it would be nice to see that cells are sufficiently distinct to be counted and the low magnification images presented do not permit this.

We increased the size of the figure and increased the contrast. The relative GFP expression was performed by quantifying the volume occupied by the GFP cells using the Imaris software after confocal scanning. Concerning the goblet cells, they were not counted on the images but directly on the microscope as it allows a better discrimination of the cells compared to the images. Indeed these images represent confocal projection images which represent the sum of all confocal sections.

8) There are problems throughout the manuscript with gene/protein/transgene nomenclature that need to be corrected.

The gene/protein/transgene nomenclature has been checked all along the manuscript.

9) The authors should stick to either “EE cells” or “EEC”.

This has been done

10) Not all panels of Figures are referred to in the text – Figure 4A, for instance. Some panels are referenced but do not exist – Figure 4H, for instance. “Fig” versus “Figure” versus “figure” needs to be homogenized.

Figure 4A (now 5A) : This image showing the rescue of the EEC by Neurod1 has been already shown in figure 1 and figure 2. It is shown in Fig 5A only to allow a direct comparison with the tg6, tg7 and tg8. Therefore it was not referred to in the text.

We corrected the annotation for figure 4H

Figure has been standardized as Fig

We hope that you will find our explanations and changes satisfactory. Thank you for all your comments that gave us a chance to improve our work.

We are looking forward to hear from you,

Sincerely yours,

Marianne Voz

1.	Casey BH, Kollipara RK, Pozo K, Johnson JE. Intrinsic DNA binding properties demonstrated for lineage-specifying basic helix-loop-helix transcription factors. Genome Res. 2018. doi:10.1101/gr.224360.117

2.	Quan XJ, Denayer T, Yan J, Jafar-Nejad H, Philippi A, Lichtarge O, et al. Evolution of neural precursor selection: functional divergence of proneural proteins. Development. 2004/04/16. 2004;131: 1679–1689. doi:10.1242/dev.01055dev.01055 [pii]

3.	Guillemot F, Hassan BA. Beyond proneural: emerging functions and regulations of proneural proteins. Curr Opin Neurobiol. 2017;42: 93–101. doi:10.1016/j.conb.2016.11.011

4.	Thambyrajah R, Ucanok D, Jalali M, Hough Y, Wilkinson RN, McMahon K, et al. A gene trap transposon eliminates haematopoietic expression of zebrafish Gfi1aa, but does not interfere with haematopoiesis. Dev Biol. 2016;417: 25–39. doi:10.1016/j.ydbio.2016.07.010

5.	Sztal TE, Stainier YR. Transcriptional adaptation: a mechanism underlying genetic robustness. Development. 2020;147. doi:10.1242/DEV.186452

6.	Lizio M, Ishizu Y, Itoh M, Lassmann T, Hasegawa A, Kubosaki A, et al. Mapping mammalian cell-type-specific transcriptional regulatory networks using KD-CAGE and ChIP-seq data in the TC-YIK cell line. Front Genet. 2015. doi:10.3389/fgene.2015.00331
