# Supplementary material for: Identification of an evolutionarily conserved domain in Neurod1 favouring enteroendocrine versus goblet cell fate
Source: PLoS Genet. 2022 Mar 14;18(3):e1010109. doi: 10.1371/journal.pgen.1010109 (PMC8959185; doi:10.1371/journal.pgen.1010109)
Supplement: Attachment — Submitted filename: 2022 02 14 reponses to the reviewers.docx [file pgen.1010109.s012.docx]

Dear Editor and Reviewers,

Please find enclosed our second revision of the manuscript entitled “Identification of an evolutionarily conserved domain in Neurod1 favouring enteroendocrine versus goblet cell fate” for further consideration at PLOS Genetics. We addressed now the concern of the first reviewer on the difficulty to interpret the histological analysis of the Neurod1^ΔUCE^ mutant by providing new images that better display the intestinal structure (revised figure 8)

**Response to the comments of the reviewers**

Reviewer #1:

Authors add histology for the neuroD1 mutant but fail to add histology from any of the experiments using the transgenic fish. Determination of whether the intestine is organized in a similar way to WT is important for interpretation of experiments. Histology added to Figure 8 for the neuroD1 mutant makes it hard to determine how the epithelium is affected. Is the epithelium still a single layer of cells in the region with additional goblet cells? It looks like the lumen may be occluded or the epithelium is stratified.

We agree with the reviewer that the histology shown on figure 8 for the Neurod1^ΔUCE^ mutant did not allow to answer the question whether the epithelium is affected. This picture was chosen as it illustrated nicely the increase of the goblet cells and the widening of the midgut of the Neurod1^ΔUCE^ mutant at 5 dpf. To evaluate whether the epithelium was stratified or not, we carefully analysed about 20 Neurod1^ΔUCE^ mutant embryos. Based on that new analysis, we can now conclude that the epithelium of the mid-intestine is still a single layer of cells with additional goblet cells. Furthermore, when we analysed successive sections of the same larvae, we could see that the lumen is not occluded. As the image of figure 8 K-M can be misinterpreted, we replaced it by another one that better shows the intestinal structure.

Inducing tg1 to tg8 in the ascl1a^-/-^ transgenic fish could indeed affect the intestinal structure. For example, inducing tg7 leads to a widening of the midgut due to the huge increase in the number of goblet cells as shown in fig 5C. But characterizing the intestine of these ascl1a^-/-^ transgenic fish by histology is a time-consuming process that would necessitate much more time than one month, which was the time allocated for the second revision of the paper. Indeed, it would require first to characterize histologically the intestine of the ascl1a-/- mutant as it was not done in our previous study of Flasse et al. (2013) nor in Roach et al. (2013). Secondly, for each transgenic line tested, we need to genotype about 100 heat-shocked larvae to sort ascl1a-/- mutant versus sibling controls. Then we must embed them, section them on a microtome, stained and analysed their histological characteristics on several sections. We believe that this aspect is beyond the scope of this paper.

Reviewer #2:

The authors have addressed the major point raised concerning the null phenotype of a neuroD1 mutant and the results are clear. As such, I have no further issues with the study.

We hope that you will find our explanations and changes satisfactory. Thank you for all your comments that gave us a chance to improve our work.

We are looking forward to hear from you,

Sincerely yours,

Marianne Voz
